# Assessing the Carboxymethylcellulose Copper-Montmorillonite Nanocomposite for Controlling the Infection of *Erwinia carotovora* in Potato (*Solanum tuberosum* L.)

**DOI:** 10.3390/nano11030802

**Published:** 2021-03-21

**Authors:** Ryan Rienzie, Lasantha Sendanayake, Devika De Costa, Akbar Hossain, Marian Brestic, Milan Skalicky, Pavla Vachova, Nadeesh M. Adassooriya

**Affiliations:** 1 Postgraduate Institute of Agriculture, University of Peradeniya, Peradeniya 20400, Sri Lanka; ryanrienzie@gmail.com (R.R.); devikadecosta@gmail.com (D.D.C.); 2 Agribusiness Centre, Faculty of Agriculture, University of Peradeniya, Peradeniya 20400, Sri Lanka; 3 Postgraduate Institute of Science, University of Peradeniya, Peradeniya 20400, Sri Lanka; lasanthasendanayake@gmail.com; 4 Department of Agricultural Biology, Faculty of Agriculture, University of Peradeniya, Peradeniya 20400, Sri Lanka; 5 Department of Agronomy, Bangladesh Wheat and Maize Research Institute, Dinajpur 5200, Bangladesh; 6 Department of Plant Physiology, Slovak University of Agriculture, Nitra, Tr. A. Hlinku 2, 949 01 Nitra, Slovakia; marian.brestic@uniag.sk; 7 Department of Botany and Plant Physiology, Faculty of Agrobiology, Food, and Natural Resources, Czech University of Life Sciences Prague, Kamycka 129, 165 00 Prague, Czech Republic; vachovap@af.czu.cz; 8 Department of Chemical & Process Engineering, University of Peradeniya, Peradeniya 20400, Sri Lanka

**Keywords:** antimicrobial, carboxymethylcellulose, Cu^2+^ ions, *Pectobacterium carotovorum*, nanoclay, nanocomposite, bacterial soft rot of potato

## Abstract

A novel antimicrobial formulation based on carboxymethylcellulose (CMC) spray-coated Cu^2+^ intercalated montmorillonite (MMT) nanocomposite material was prepared and its morphology, internal structure, and bonding interactions were studied. Meanwhile, the antibacterial efficacy and release behavior of Cu^2+^ was also determined. PXRD patterns indicated the intercalation of Cu^2+^, while FTIR spectra and TGA traces confirmed the association of Cu−MMT with CMC. SEM study revealed the improvement of nanocomposites by CMC, without disturbing the clay structure. TEM and EDAX studies indicated the distribution of Cu (copper) throughout the composite. *In vitro* antibacterial assays performed with *Erwinia carotovora* revealed effective bacterial growth suppression, indicating the potential of this material in controlling soft rot of potatoes (*Solanum tuberosum*); also observed was a connection between growth inhibition and concentration of CMC spray coats indicating a positive relationship between Cu^2+^ release and concentration of the CMC coatings. The activity pattern of the nanocomposite displayed a significant degree of sustained-release behavior.

## 1. Introduction

Nanoscience-based products and applications have fetched a greater attention in a range of fields including the biocide industry. Cu is a major active ingredient of chemicals with cidal effects because it is effective against numerous plant and mammalian diseases [1,2]. The use of Cu in agricultural applications goes back to the 19th century. Inorganic Cu compounds with fungicidal and bactericidal properties, and as fertilizer additives are very popular due to their low cost [3]. Another advantage possessed by copper is that bacteria and fungi cannot build up resistance against it as they do with antibiotics and synthetic fungicides that are organic in origin. Among the Cu based biocides, Cu(NO_3_)_2_, CuSO_4_.5H_2_O, Cu(OCl)_2_, Cu(OH)_2_, and Cu_2_O are more common, with the metallic copper content of commercial formulations being generally in the range of 5–85% [4]. In bacterial cells, Cu^2+^ causes membrane damage and loss of cell integrity.

Bacterial diseases have gained greater attention in crop agriculture. Soft rot caused by *Erwinia* sp. is one of the serious diseases that affect many agriculturally important crops [5,6,7]. *Erwinia carotovora* subsp. *atroseptica* van Hall [8] and Dye [9,10,11] and *Erwinia carotovora* subsp. *carotovora* Jones [12]; Bergey et al. [13] are considered as important plant pathogenic species of the genus *Erwinia* [5], and these subspecies are considered as the main source of primary inoculum for soft rot of potato (*S. tuberosum*) [5]. Lack of effective control strategies to protect potatoes in the field or during storage has hindered all efforts at inoculation, and therefore the use of non-contaminated planting material has been identified as one of the better control strategies [14].

Recent research showed that by planting cut potato tuber pieces of 60 g each (optimum size), it is possible to generate better harvests with very high marketable yields in Sri Lanka [15]. However, cutting the tubers results in exposing a considerable surface area of wounded tissues that attract plant pathogenic fungi and bacteria. Therefore, in general, it is advised to store the cut tubers for a couple of days to allow them to develop the corky layer that prevents infection by pathogens; this is followed by dipping the pieces in a suitable fungicide before planting in the soil. Whereas there is a remedy for fungi by treating the planting material with fungicides, there is no particular method to control bacteria such as *Erwinia*.

The uncontrolled release into the environment after the application is a major problem associated with the use of most pesticides. This results in the accumulation of toxic residues and/or heavy metals in the soil and water bodies [16]. In general, 60–70% of the pesticides do not reach the target objects and are removed through leaching, volatilization, immobilization, and erosion [17]. Some of these pollutants are heavy metal-based chemicals that tend to accumulate in the environment, with Cu being one such important heavy metal [18,19,20]. The presence of excess Cu has detrimental effects on many organisms as has been emphasized by many researchers [21,22,23,24,25]. However, copper is considered less toxic to humans compared to other heavy metals like cadmium, lead, and mercury [26]. All the same, improving release properties through the formulation of any pesticide plays a major role in combatting such negative impacts. Among such attempts, incorporation of clay and/or polymer matrices together with the active ingredients is one of the widely researched methods. 

Clay minerals possess good adsorption properties, cation exchange capacity (CEC) and also facilitate controlled delivery [27]. Antibacterial activity of metal exchanged montmorillonite composites such as Cu−MMT [26,27,28,29,30,31,32,33], Ag-MMT [29,34], and Zn−MMT [29] have been studied by various researchers. MMT-metal ion composites associated with polymers or resins have also been synthesized and tested [35]. Additionally, other Cu forms such as CuO [36,37] and Cu_2_O [38], intercalated with MMT have also been studied for the same purposes. However, these studies were based on human and animal pathogenic bacteria like *Escherichia* spp., and mesophilic bacteria—such as *Pseudomonas* spp., *Streptococcus* spp., and *Salmonella* spp.—and not on plant pathogenic bacteria.

Through the current study, carboxymethylcellulose (CMC) spray-coated Cu^2+^ intercalated, MMT nanocomposites were prepared and the prepared nanocomposites were fully characterized in terms of their internal structural properties and morphology using powder X-ray diffraction (PXRD), Fourier transformed infrared spectroscopy (FTIR), thermal analysis using thermo gravimetric analysis (TGA), scanning electron microscopy (SEM), and transmission electron microscopy (TEM). In addition to that, *in-vitro* antibacterial efficacy against *Erwinia carotovora* was also determined using two experiments. Finally, the release behavior of one composite was studied in sandy soil. CMC was selected because it is a biodegradable polymer that can trigger the sustained release of active ingredients. Employing biodegradable polymers such as carboxymethylcellulose has drawn significant attention compared to synthetic petroleum-derived polymers because biodegradable polymers are less toxic, biocompatible, and biodegradable. Studies have claimed that polymer encapsulated formulations are more effective in terms of extended activity than non-encapsulated forms as encapsulation reduces the losses caused by unwanted release [39] whilst the clay component in the composite improves the structural properties of the nanocomposite [40].

## 2. Materials and Methods

### 2.1. Preparation of Cu^2+^ Exchanged MMT (Cu-MMT)

A portion of 9 g of Na−MMT (Sigma Aldrich, St. Louis, MO, USA) were weighed and mixed with 200 mL of 0.05 M CuSO_4_.5H_2_O (Sigma-Aldrich) solution at room temperature (25 °C) and after stirring the mixture was kept overnight. The resulting solution was centrifuged for 20 min at 5000 rpm. Then it was decanted, and the remaining solid material was washed three times with distilled water. Following this, the material was oven-dried for 6 h at 80 °C. After drying it, a sample of the Cu-MMT was analyzed for the percentage of copper by weight using a Thermo Scientific ICE 3500 atomic absorption spectrophotometer (AAS).

### 2.2. Preparation of CMC Spray-Coated Cu-MMT Nanocomposites

CMC (CDH laboratories, New Delhi, India: Viscosity 1% at 25 °C, 1200–2400 cps) at different concentrations, specifically 2.5, 5 and 7.5 g/L were prepared using distilled water heated up to 80 °C. After adding CMC, the solution was continuously stirred for 2 h and then cooled down to room temperature. The three solutions at room temperature were poured into a spray gun in turn. Prepared Cu−MMT from each composite was spread on Petri dishes (2.5 g each) uniformly. Each Petri dish with Cu−MMT was sprayed with different strengths of the CMC solutions while slightly shaking the dishes. After spraying, the composites were kept in a drying oven at 60 °C for 6 h to remove moisture.

### 2.3. Characterization

#### 2.3.1. Structural Properties

PXRD patterns of all synthesized samples were recorded using a Bruker D8 Focus X-ray powder diffractometer using Cu K radiation (=0.154 nm) over a 2θ range of 3–65° with a step size of 0.02° and a step time of 1s. The nature of chemical bonding of the synthesized samples was determined using a Bruker Vertex 80 FTIR spectrometer, by spanning the range from 600 to 4000 cm^−1^ using the attenuated total reflectance technique. The thermal behavior of the synthesized samples was studied by performing TGA (TA Instruments SDTQ600). The samples (10–15 mg) were heated from ambient temperature to 100 °C (ramp 10 °C/min) in a nitrogen environment (100 cm^3^/min N_2_ flow rate). The particle size and the morphology of the synthesized samples were studied using a HITACHI SU6600 SEM and Philips CM30 TEM and energy dispersive X-ray analysis (EDAX).

#### 2.3.2. Antibacterial Properties

##### Experiment 1: Bacterial Growth Inhibition Test

*In-vitro* antibacterial activity was assessed using gram-negative bacterium *Erwinia carotovora*, which was isolated from potatoes showing soft rot symptoms which were collected from a field in Nuwara-Eliya, Sri Lanka. A single bacterial colony of *E. carotovora* obtained through sub-culturing was inoculated into 50 mL of nutrient broth (Sigma-Aldrich) and shake-incubated at 30 °C for 72 h. Then the bacterial cell concentration of the culture was determined by the dilution plate technique. At the same time, Petri dishes with nutrient agar were prepared and wells were bored in the middle of the solidified agar plates. Each well was incorporated with composites of 20, 40, or 60 mg and the wells were again filled with nutrient agar. The composites were exposed to UV light for a period of 6 h before adding them into the petri dishes. Then 100 µL of the *E. carotovora* culture was spread on the surface of the material in the Petri dishes and incubated at 29 ± 1 °C for 24 h. After the incubation period, the diameter of the inhibition zones corresponding to each weight level was measured using a Vernier caliper. The experiment was replicated thrice and the average values of the diameters of the inhibition zones were obtained.

##### Experiment 2: Potato Tuber Inoculation Test

Certified, disease-free potatoes (variety Granola) were used for inoculation. First, the tubers were thoroughly washed with water and then surface sterilized with NaOCl 1% solution. After that, the outer peels were removed. Following that, they were sliced into pieces measuring approximately 2.5 × 2 × 0.5 cm^3^, which were considered as replicates. Each treatment was comprised of 10 replicates of potato pieces weighing approximately 60 g each (optimum economic size of cut potato that can be used as planting material, according to Mayakaduwa et al. [15].

The following treatments were done using all three CMC spray-coated Cu−MMT nanocomposites (2.5, 5, and 7.5 g/L CMC). The potato pieces in each replicate were treated with either 20 mg, 40 mg, or 60 mg of each composite (approximately 2 mg, 4 mg, or 6 mg/replicate). As control one group was treated with only *Erwinia*. In each of the above treatments, every replicate was inoculated with 10 µl of 1.2 × 10^5^ CFU/mL *Erwinia* cell suspension. All inoculations were done under aseptic conditions. After three days of inoculation, observations were made and the areas of infection were measured, from which the percentages of infection were calculated using the formula, [(area infected/ total area of the tissue piece) × 100] and the values were averaged.

A factor-factorial analysis was performed to determine the effect of concentration of CMC spray coating and weight of nanocomposites on the percentage of infection in the above two experiments.

#### 2.3.3. Soil Release Study

**Figure 1:**
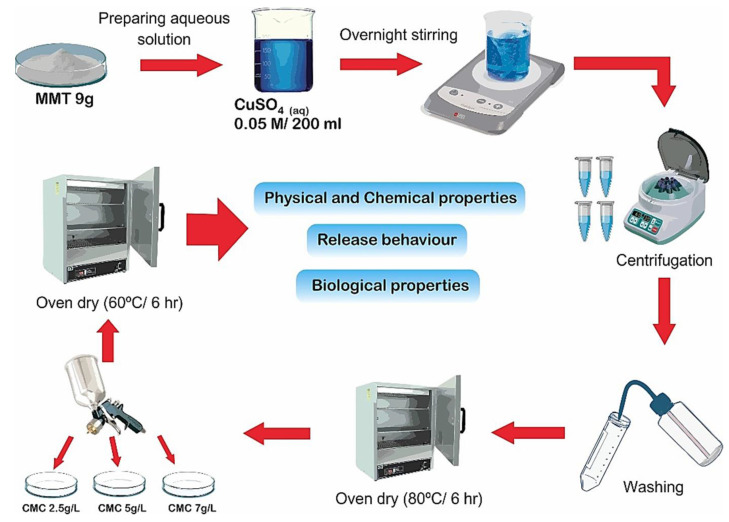
Schematic representation of the experimental procedures.

A soil release study was conducted as in soil medium, an interaction takes place between the tubers treated with composites and soil. Accordingly, One Cu−MMT−CMC composite (Cu−MMT−CMC 5.0 g/L) out of three was tested for its release behavior and the release of Cu was compared with pure CuSO_4_.5H_2_O and uncoated Cu-MMT. Cu−MMT−CMC 5.0 g/L was selected as it was stable in the presence of atmospheric water. The test was conducted using two potato growing soil types (i.e., sandy soil with low organic matter content and loamy soil with high organic matter content) packed in two leaching columns for seven days. The leachates were analyzed for the quantification of Cu by means of atomic absorption spectrophotometry (AAS) using a Thermo Scientific ICE 3500 spectrophotometer. The data were plotted as the cumulative percent release of Cu over time (days). A schematic representation of all experimental procedures is provided in Figure 1.

## 3. Results and Discussion

### 3.1. Structural Properties

#### 3.1.1. PXRD Characterization

**Figure 2:**
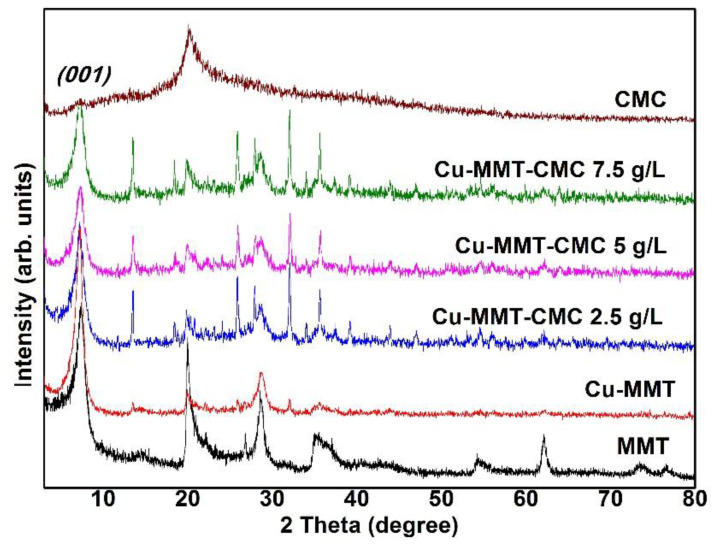
PXRD patterns of Na−MMT, CMC, Cu−MMT, and Cu−MMT−CMC nanocomposites.

The PXRD results provide information about the purity of materials and the crystalline phases present in the prepared nanocomposite. PXRD patterns for MMT, CMC, and Cu−MMT−CMC nanocomposites are shown in Figure 2. Basal d_spacing_ values were determined using X-ray diffraction. Accordingly, Na-MMT showed a diffraction peak at 2θ = 7.40°: 1.19 nm (corresponding interlayer d_001_ value according to the Bragg equation). Na^+^ ions present in the interlayer space of MMT exchange with Cu^2+^ ions, causing the d_001_ value to shift to 1.23 nm as hydrated Cu^2+^ ions cause the interlayer distance to increase [41]. Many researchers have reported d_spacing_ changes in Cu-MMT composites with values ranging from about 1.21 to 1.27 [22,28,31,42,43,44,45,46,47,48]. Small new reflections at 2θ = 13 in the diffraction patterns of Cu-MMT could be due to amorphous Cu(OH)_2_. H_2_O [44] while d_spacing_ of clays vary with the level of hydration. After spray coating CMC on Cu−MMT particles, interlayer space remains unchanged as it is a surface coating on the solid Cu-MMT particles. It is important to mention that the spray coating of CMC was preferred over the direct addition of Cu−MMT into the solution as the acidity of Cu^2+^ ions might lead to coagulation of CMC.

#### 3.1.2. FTIR Analysis

**Figure 3:**
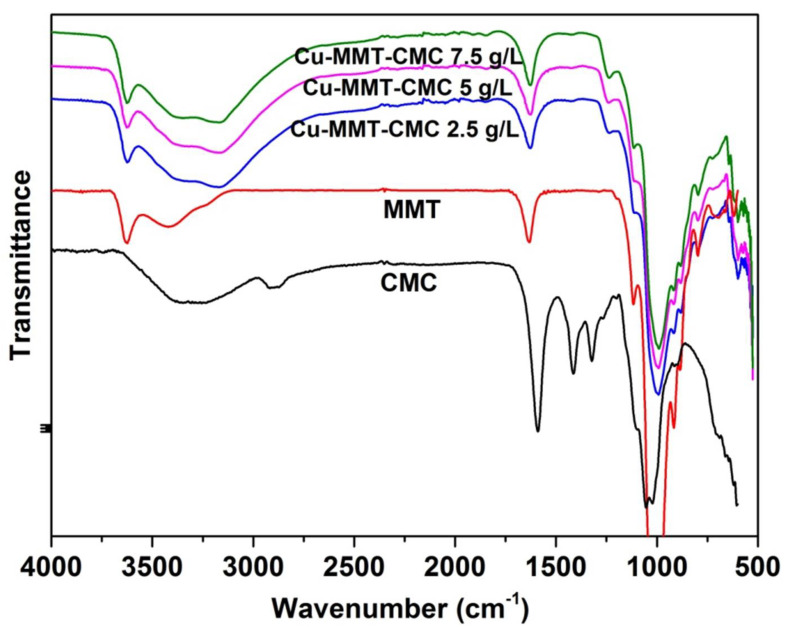
FTIR spectra for Cu−MMT−CMC nanocomposites.

FTIR technique is used to study the bonding interactions involve within the nanocomposite as intermolecular bonds play an important role in the release kinetics of active ingredients present in the prepared nanocomposite. FTIR spectra for MMT, CMC, and Cu−MMT−CMC are shown in Figure 3. CMC shows a band at 2908 cm^−1^ due to C–H stretching of the –CH_2_ groups and the band due to ring stretching of –COO^−^ appears at 1600 cm^−1^. In addition, the bands in the region 1350–1450 cm^−1^ are due to symmetrical deformations of -CH_2_ and -COH groups. The bands due to –CH_2_OH stretching mode and -CH_2_ vibrations appear at 1070 and 1020 cm^−1^, respectively [49]. MMT shows a characteristic absorption band at 3400 cm^−1^ due to the O-H stretching of adsorbed water and the shoulder at 3628 cm^−1^ due to structural -OH groups of MMT. Peaks are seen at 995 cm^−1^ and 1125 cm^−1^ are due to Si-O stretching vibrations of MMT layers [47]. In the FTIR spectrum of Cu−MMT−CMC, O-H stretching of adsorbed water of MMT shows two lobes at 3350 and 3170 cm^−1^. In addition, the shoulder at 3628 cm^−1^ due to structural OH of MMT has shifted slightly to 3622 cm^−1^.

#### 3.1.3. Thermal Analysis

**Figure 4:**
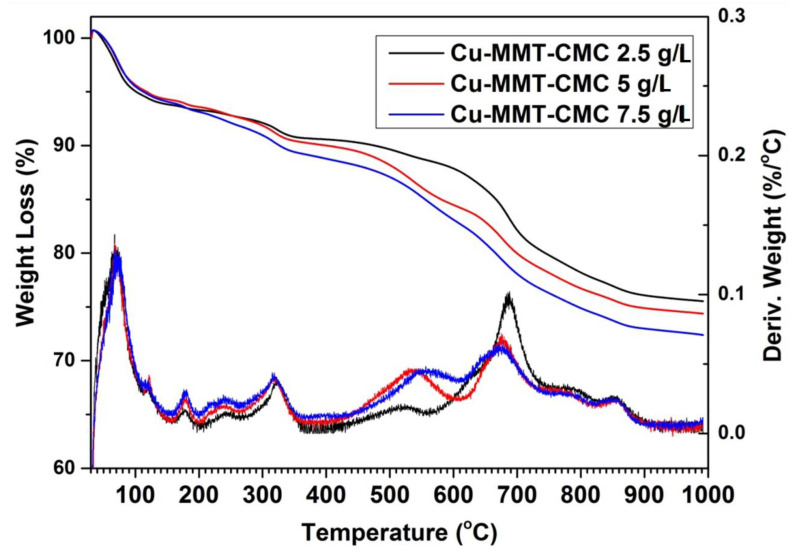
TGA (which denotes weight loss) and DTA (which denotes derivative weight) profiles for Cu−MMT−CMC nanocomposites.

The thermal analysis provides the thermal stability of the materials prepared. Here the weight change of the material is determined by heating the sample at a constant rate. TGA and differential thermal analysis (DTA) traces for Cu−MMT−CMC nanocomposites are shown in Figure 4. All three Cu−MMT−CMC nanocomposites show similar thermal events. Up to 200 °C, a total weight loss of approximately 15% occurs due to dehydration of surface absorbed water in CMC and MMT in the nanocomposite. Furthermore, at around 250 °C, a 2% weight loss occurs due to the removal of water of the crystallization of CuSO_4_. The thermal event around 300 °C is due to the degradation of CMC [47] in the composites and the losses are in keeping with the concentration of CMC sprayed onto the composites. For instance, the 2.5, 5, and 7.5 g L^−1^ concentration levels show 2%, 3%, and 4% of weight loss, respectively. The thermal peak at 500 °C is due to further degradation of CMC residues. Those losses are approximately 3%, 5%, and 6% and correspond to the concentrations of 2.5, 5, and 7.5 g/L, respectively. The prominent thermal event at around 600–700 °C is due to the collapse of the layered structure of MMT [47]. At 800 °C another thermal event could be observed due to the removal of SO_2_ and O_2_ from CuSO_4_ leaving behind CuO. Finally, at 1050 °C another thermal event occurs due to the removal of O_2_ from CuO leaving a residue of Cu_2_O (Figures S1 and S2).

#### 3.1.4. Microscopy

##### Scanning Electron Microscopy (SEM)

**Figure 5:**
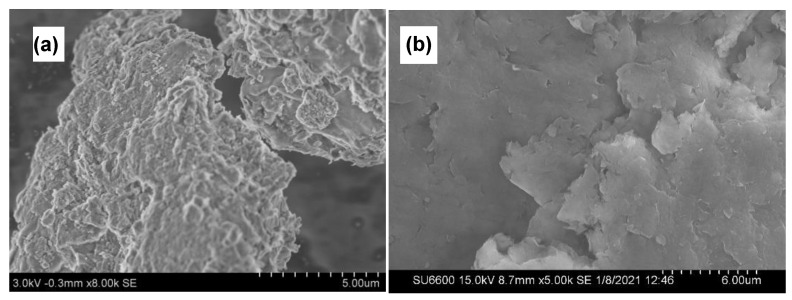
SEM images of Cu−MMT−CMC nanocomposites (**a**,**b**).

SEM technique is used to obtain information on the surface topography of prepared nanocomposites. As seen in Figure 5a,b SEM images show that the plate-like layered structure of MMT continues to remain even after the formation of the composite which facilitates the controlled release behavior of active ingredients present in the nanocomposite. Furthermore, no accumulations of CMC could be seen, confirming the relative uniformity of the spray coats.

##### Transmission Electron Microscopy (TEM) and Energy Dispersive X-ray Analysis (EDAX)

**Figure 6:**
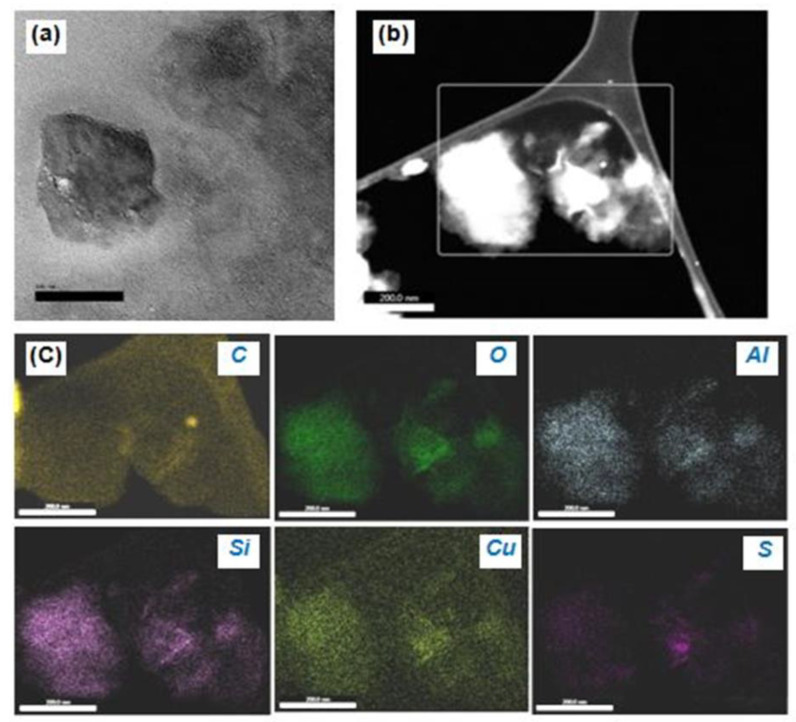
(**a**) TEM image of Cu−MMT−CMC nanocomposite, (**b**) area considered for EDAX analysis, and (**c**) EDAX elemental mapping images showing the existence of MMT layers composed of C, O, Al, Si, Cu, and S within the Cu−CMC−MMT nanocomposite.

TEM is vital for the study of the internal features of a material. Also, EDAX provides chemical information about the material. The nanoscale Cu−MMT−CMC nanocomposite can be seen in Figure 6a as having a mixed morphology of a large intercalated tactoid and small intercalated tactoids of MMT clay dispersed throughout the polymer under a magnification of 200 nm. This high magnification shows a large tactoid due to the flocculation of clay layers and a region of small intercalated tactoids together with a few intercalated layers. Figure 6b shows the Cu particles intercalated in MMT layers along with the CMC polymer in the form of a network in a dark field TEM image.

Besides, Figure 6c shows a nanoscale TEM−EDAX elemental mapping in which MMT layers consisting of O, Al, and Si were detected as the primary component of the MMT while CMC polymer consisting of C was detected along with the C which emanates from the sample substrate. S was observed in varying proportions throughout the nanocomposite which arises from sulphate residues present in the nanocomposite. As seen in Figure 6c TEM−EDAX image of Cu shows that Cu is distributed more evenly over the MMT layer compared to other elements.

### 3.2. In Vitro Antibacterial Activity Against Plant Pathogenic Erwinia carotovora

#### 3.2.1. Experiment 1: Bacterial Growth Inhibition Test

**Figure 7:**
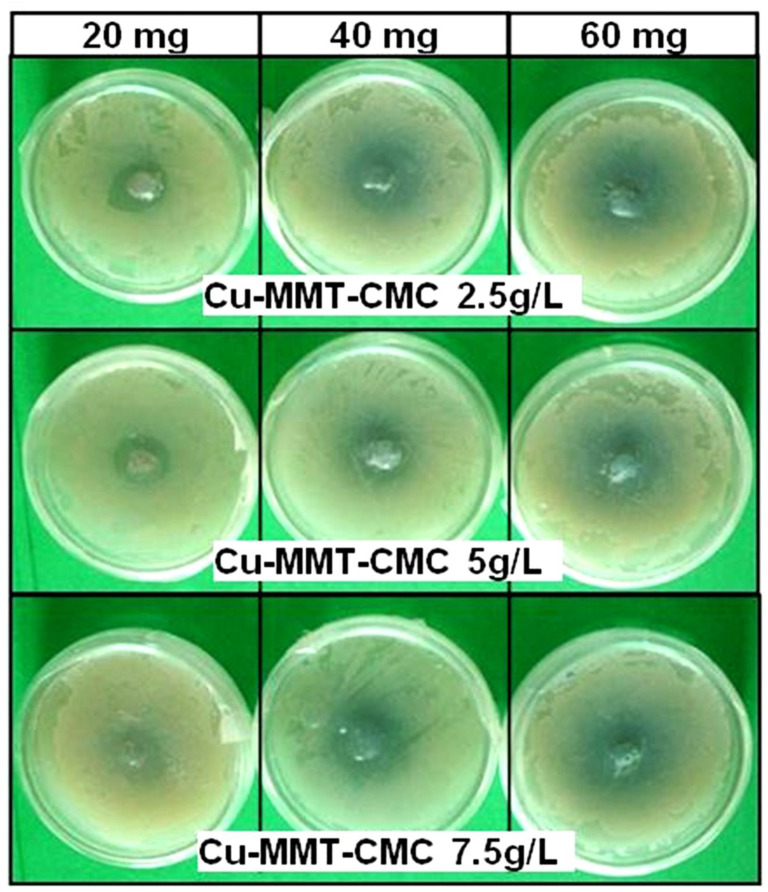
Antibacterial activity of Cu−MMT−CMC nanocomposites.

The cell concentration of the bacterial culture was determined as 3 × 10^9^ CFU/ mL through the dilution plate technique. Figure 7 shows the results of a bacterial inhibition test for all three Cu−MMT−CMC nanocomposites whereas Table 1 shows the diameters of the inhibition zones observed. The results clearly indicate that the diameter of the inhibition zone increased with the number of composites tested, indicating the antibacterial activity of all types of nanocomposites used in the study against *E. carotovora*.

The output of factor-factorial analysis can be interpreted in terms of a particular weight level if a slight reduction of the diameter of the inhibition zone can be observed when the concentration of the CMC spray coat increases. An interaction effect between factors was not significant when the *p*-value was 0.102 (*p >* 0.05). However, the main effects, the specifical concentration of spray coat and weight were highly significant correspondingly with *p =* 0.006 and *p =* 0.000 values (*p <* 0.05) to the diameter of the inhibition zone.

This indicates Cu^2+^ ions can be released into the medium by penetrating through the polymer network of the medium. Therefore, it can be inferred that the mobility of Cu^2+^ is more or less in step with the current range of concentration. Compared to the polymer-coated nanocomposites, the negative controls, i.e., CMC, MMT (Na^+^), or MMT−CMC did not show any antibacterial activity (data not shown) against *E. carotovora*.

#### 3.2.2. Experiment 2: Potato Tuber Inoculation Test

The percent infection was decreased with the increased concentration of the CMC spray coats and increased weight of nanocomposites applied to potato tuber pieces while the control pieces showed higher infection percentages compared to other treatments (Table 2; Figure S3). Here the Cu−MMT−CMC 2.5 g/L (60 mg) showed the lowest percentage of infection. Through the factor-factorial analysis, it was revealed that the concentration of the spray coat has a significant effect on percentage infection with the *p*-value of 0.0016 (*p <* 0.05). Similarly, the weight of the nanocomposite also showed a significant effect on the percentage of infection (*p <* 0.001). Moreover, the interaction effect of concentration of the spray coat and weight of the nanocomposite was also significant as *p*-value was 0.0057 (*p <* 0.05).

Cu based compounds have been proven to be efficacious against many plant pathogenic bacteria including *Erwinia* spp. [50]. Cu compounds are widely used against many *Erwinia* spp. including *E*. *amylovora* in apple and pear, *E*. *mangiferae* in mango and *E. trachiphila* in cucurbits [51]. Among the Cu based compounds, CuSO_4_.5H_2_O is in common usage for controlling plant pathogenic bacteria [23]. *In-vitro* experimental results have proven its ability to control plant pathogenic bacteria species belonging to the genus *Pectobacterium* (formerly in the genus *Erwinia*) [52].

Interestingly, with respect to the potato crop, Abo-Elyousr et al. [5] have demonstrated that spraying CuSO_4_ had the highest controlling effect on soft rot caused by *E. carotovora*. In addition to that, Zhang et al. [53] reported that potato soft rot caused by *E. carotovora* can be effectively controlled by using CuSO_4_. Furthermore, Gracia-Garza et al. [54] demonstrated that treatment of greenhouse-grown calla lilies (*Zantedeschia* sp.) with sub-irrigation laced with Cu and Cu (OH)_2_ reduced the soft rot incidence of *E. carotovora* without compromising the plant growth.

Several other types of bacteria besides the one tested in the present study were studied. For instance, Cu−MMT composites were prepared and tested with *E. coli* and it was found they suppressed the growth of this organism [27,28,31,32,33]; this showed that the efficaciousness and ability of Cu-MMT were superior to Na-MMT [55]. Ag-MMT was also tested against mesophilic bacteria and bacteria present in lactic acid by Costa et al. [34] and against *E. faecium* by Magaña et al. [30], which showed it to be efficacious in all these cases. MMT exchanged with Cu, Ag, and Zn, when tested against *E. coli*, *Pseudomonas ostreatus* and *P*. *cinnabarinus* had shown promising results in respect of suppression of growth of those bacteria [29]; in the same study, it was reported that Cu−MMT is efficacious over Cu^2+^. Furthermore, certain studies had reported that Cu^2+^ intercalated MMT can bind with polymers to enhance the sustained release nature of Cu^2+^ into the medium and act against *E. coli* and *S. aureus* effectively. For example, this led to the development of a Cu−MMT-epoxy resin [35]. Furthermore, epoxy matrices with CuO nanoparticles embedded in MMT [37] and Cu_2_O embedded in octadecyl amine-modified MMT [21] have also been studied and found to be very effective at suppressing the growth of *E. coli*. Therefore, the present study provides new insights into employing Cu−MMT-polymer composites for controlling plant diseases.

In general, positively charged biomaterial—i.e., polymer surfaces show an antimicrobial effect on adhering Gram-negative bacteria [56]. Replacement of Na^+^ in Na−MMT by Cu^2+^ increases the presence of positive charges on the surface and in the interlayer space of MMT where the Gram-negative bacteria tend to get attached. *E. carotovora* is a Gram-negative bacterium that is highly vulnerable to Cu^2+^ [29,31]. The addition of a biopolymer like CMC on Cu−MMT particles further enhances the release of Cu^2+^ ions by absorbing moisture from the environment. Moisture absorption by CMC due to its highly hydrophilic carboxylic group causes the CMC composite to swell to a marked extent, depending mainly upon the pH of the medium [57,58]. The MMT also increases the swelling capacity of certain composites, i.e., CMC-g-poly (acrylamide)-MMT [59].

In Sri Lanka, losses caused by *E. carotovora* are significant in certain economically important crops [60,61]. As observed, farmers in Sri Lanka tend to cut the seed potato instead of the whole tuber to minimize the costs incurred. Under such situations, infestations could be higher as the cut surfaces facilitate the penetration process. Furthermore, Cu^2+^ is not only efficacious against bacteria, but it also affects soil-borne fungi such as *Phytopthora* spp. Its broad range of action against organisms that destroy crops has favored higher usage of Cu^2+^ throughout the world.

### 3.3. Soil Release Study

**Figure 8:**
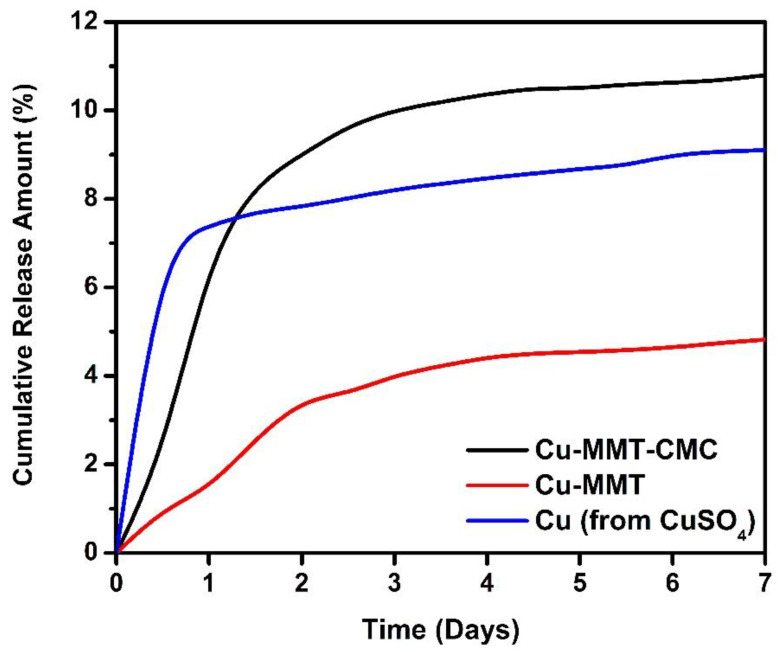
Cumulative release of Cu^2+^ from Cu−MMT−CMC 5.0 g/L, Cu−MMT, and CuSO_4_.5H_2_O.

The cumulative amounts of Cu released plotted against time from CuSO_4_.5H_2_O, Cu−MMT, and Cu−MMT−CMC were measured using a sandy soil sample. The results are shown in Figure 8. Overall, the Cu releasing pattern from pure Cu (from CuSO_4_) and Cu−MMT nanocomposite are similar when compared to Cu−MMT−CMC nanocomposite, which nearly leveled off after the third day.

As shown in the results, at the end of the first 12 h, Cu−MMT−CMC cumulative release percentage was around 2.2. The release percentage gets gradually increased to five times of initial quantity until day 5 and then gets leveled off just over 10.5%. Initially, there is a 1% of Cu release from Cu-MMT and then slightly increased release behavior up to 4.5% of Cu at 120 h which is similar to the Cu−MMT−CMC nanocomposite. Nearly two times lower release of Cu from Cu-MMT than Cu−MMT−CMC may be due to the non-covalent intercalation of Cu inside the MMT layers that induces the slow release even though they had similar kinetics. The burst release of Cu−MMT−CMC nanocomposite (coated) compared to Cu−MMT can be due to CMC coating. CMC absorbs and retains water which swells to facilitate dissolution of more trapped Cu within MMT and after that release through diffusion to the surrounding.

Based on the results obtained for pure Cu, the instantaneous Cu release was observed on the first 12 h which is around 7%. Despite this, the rest release pattern of pure Cu showed moderately increasing behavior reaching nearly 9% around day 7. However, the comparative analysis indicated that the nanocomposite had a slow-release behavior followed by instantaneous release due to the absence of modifications compared to the other two formulations. Thus, it reveals that the addition of CMC to the Cu−MMT (Cu−MMT−CMC) nanocomposite might concentrate much of the copper inside nanocomposite giving the ability to release Cu in a gradually increasing manner and the same time Cu ions which have been loaded to the intercalated MMT(Cu−MMT) layers enabling them to be released in a slow and controlled manner. In general, the release pattern of Cu from Cu−MMT−CMC gives a better and controlled release behavior in comparison to the other two forms.

In contrast, the leachate of potato soil with higher organic matter content did not contain Cu. Thus, releasing around 10% of Cu from the composite incorporated into the sandy soil is considerable. The reasons behind on not containing Cu in the leachate might be the complexation and coagulation of Cu with humic acids [62], which might be an underlying cause that prevents releasing Cu in this case.

## 4. Conclusions

Through the current work, Cu intercalated montmorillonite nanocomposites coated with three different concentration of CMC spray were formulated and characterized for their essential properties. FTIR spectra and TGA traces confirmed the association of Cu−MMT with CMC. The plate-like appearance of MMT was maintained even after nanocomposite formation which is vital for controlled release behavior. All three Cu−MMT−CMC nanocomposites showed antibacterial activity against *E. carotovora* making them suitable for tuber treatments. Furthermore, in the agar plate experiment, antimicrobial activity was slightly reduced when the concentration of the CMC spray coats increased; this implied that the Cu^2+^ ions released into the medium by penetrating through the polymer network were slightly affected by the CMC coating on the Cu−MMT particles. When the composite was tested for the release of Cu^2+^, it was found that it could release Cu^2+^ instantaneously, followed by a controlled release pattern. Furthermore, it carries a great commercial potential as clays and biodegradable CMC are environmentally friendly, biocompatible, and economically viable.

## Figures and Tables

**Table 1:** Diameters of the inhibition zones corresponding to various amounts of composites

Weight Per Well	20 mg	40 mg	60 mg
Composite *	Diameters of the inhibition zones (cm)		
C1	1.75 (±0.0115)	2.40 (±0.0)	2.50 (±0.153)
C2	1.75 (±0.116)	2.36 (±0.116)	2.42 (±0.300)
C3	1.71 (±0.010)	2.15 (±0.0115)	2.20 (±0.015)

Prior to the antibacterial test, the average Cu content of the composites in triplicate was determined through AAS and found to be 9.2%, 8.6% and 8.2% of Cu by weight respectively, which corresponded to spray coats of 2.5, 5.0 and 7.5 g/L (C1 = Cu−MMT−CMC 2.5 g/L, C2 = Cu−MMT−CMC 5.0 g/L, C3 = Cu−MMT−CMC, 7.5 g/L). Values given in parentheses in the table include standard errors of means.

**Table 2:** Variation of mean percentages of infection by *E. carotovora* on potato tuber pieces under different treatments

Treatments	Composite Weight Levels		
	20 mg	40 mg	60 mg
Composite	Mean percentage infection		
C1	0.73 (±0.168)	1.68 (±0.191)	0.18 (±0.267)
C2	2.24 (±0.223)	2.12 (±0.852)	0.46 (±0.329)
C3	2.46 (±0.223)	2.26 (±0.174)	0.56 (±0.527)
Control	2.51 (±0.142)	2.51 (±0.142)	2.51 (±0.142)

C1 = Cu−MMT−CMC 2.5 g/L, C2 = Cu−MMT−CMC 5.0 g/L, C3 = Cu-MMT-CMC, 7.5 g/L; Control: Untreated potato + *E. carotovora*; Values given in parentheses in the table include standard errors of means.

## Data Availability

Most of the recorded data are available in Tables and Figures of the manuscripts.

## Supplementary Materials

The following are available online at https://www.mdpi.com/2079-4991/11/3/802/s1, Figure S1: TGA curve of MMT; Figure S2: TGA curves of CMC; and Figure S3: TGA curves of CMC.
