# Supplementary material for: Assessing the Carboxymethylcellulose Copper-Montmorillonite Nanocomposite for Controlling the Infection of Erwinia carotovora in Potato (Solanum tuberosum L.)
Source: Nanomaterials (Basel). 2021 Mar 21;11(3):802. doi: 10.3390/nano11030802 (PMC8004016; doi:10.3390/nano11030802)
Supplement: Supplementary file 1 [file nanomaterials-11-00802-s001.pdf]

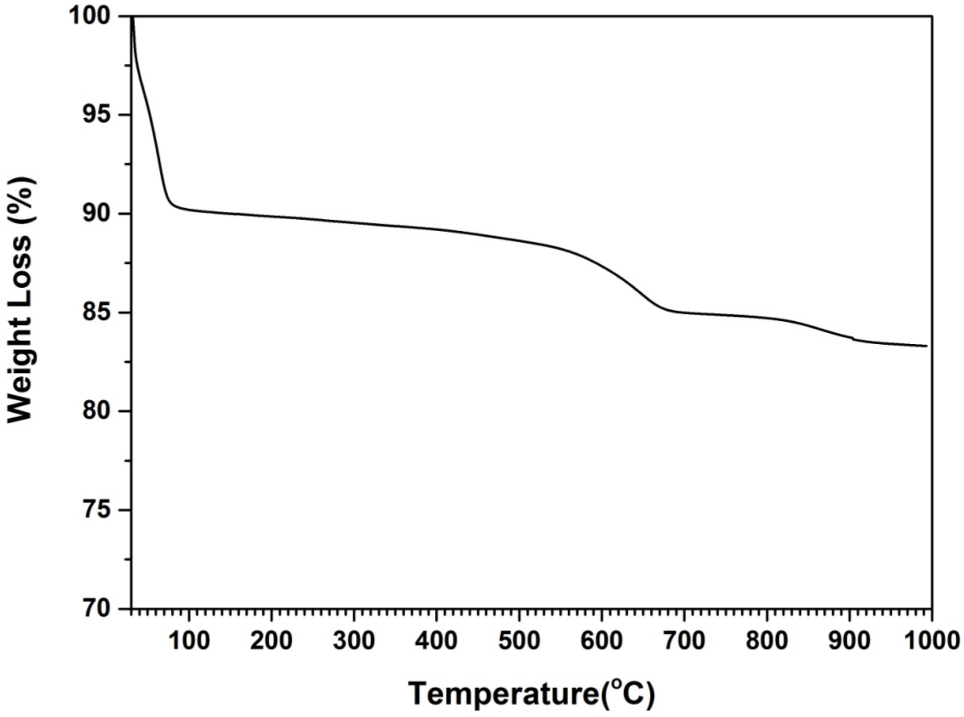

Figure S1. TGA curve of MMT

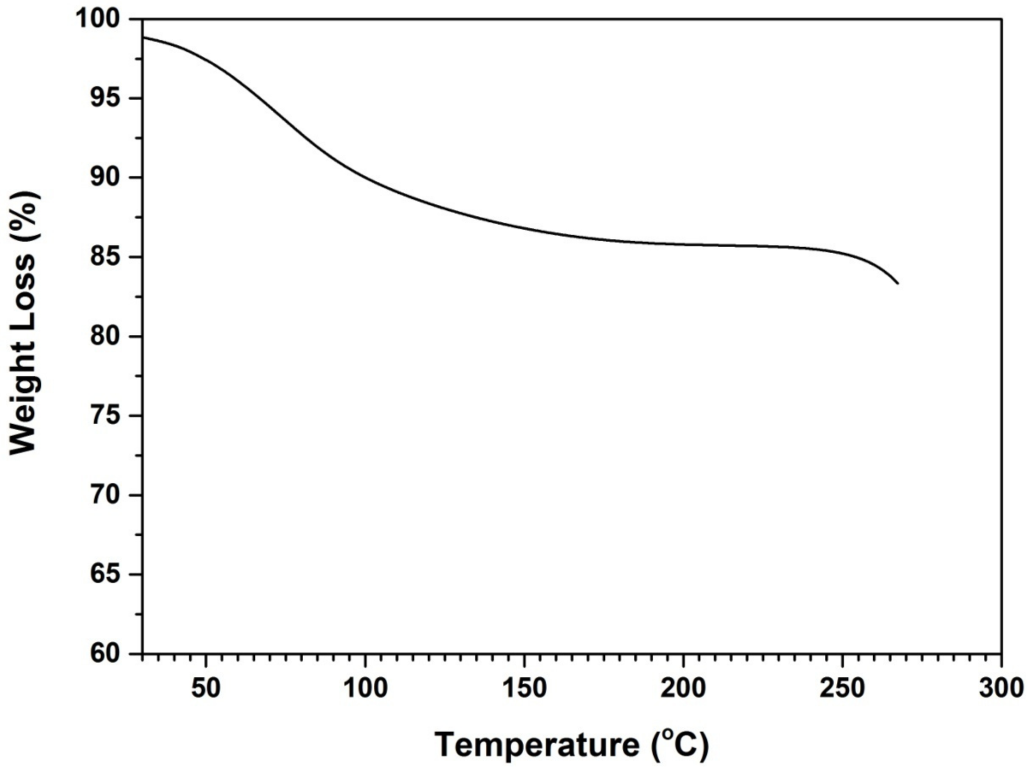

Figure S2. TGA curves of CMC

	20 mg	40 mg	60 mg
2.5 g/ L CMC	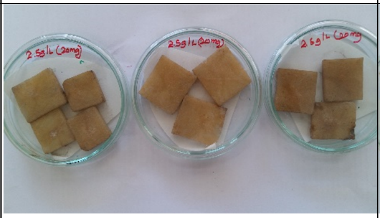	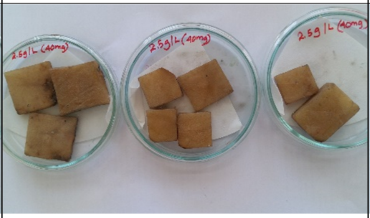	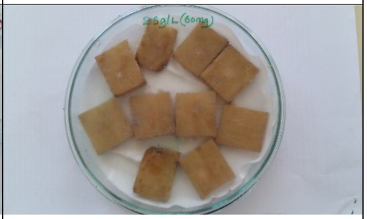
5.0 g/ L CMC	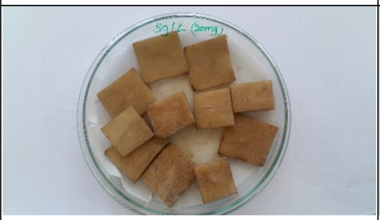	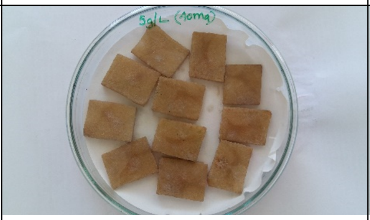	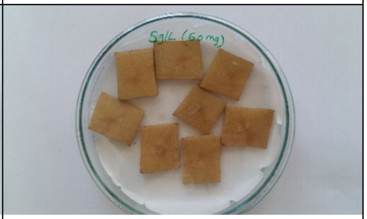
7.5 g/ L CMC	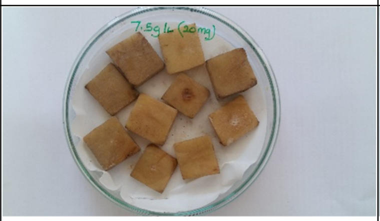	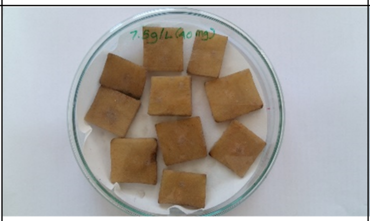	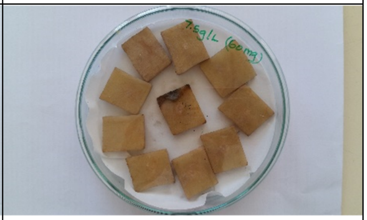
Control	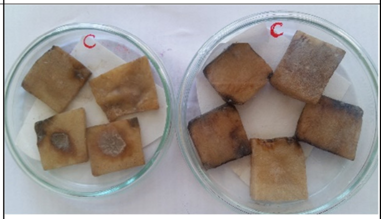		

Figure S3. TGA curves of CMC
